# Commentary: *Staphylococcus aureus* Membrane-Derived Vesicles Promote Bacterial Virulence and Confer Protective Immunity in Murine Infection Models

**DOI:** 10.3389/fmicb.2018.02346

**Published:** 2018-10-01

**Authors:** Simon Heilbronner

**Affiliations:** Department of Infection Biology, Eberhard Karls Universität Tübingen, Tübingen, Germany

**Keywords:** extracellular vescicles, *S. aureus*, immunoglobulin binding proteins, peptidoglycan, membrane

The production of extracellular vesicles (EV) by bacteria has gained growing attention in the recent years. EVs produced by the Gram-positive pathogen *Staphylococcus aureus* are currently studied by several independent groups in various countries. A number of publications is now available reporting consistently that EVs are produced by different *S. aureus* strains and contain a diverse set of proteins such as cytosolic proteins, membrane proteins, cell wall-associated proteins (CWAs) and even proteins supposed to be secreted into the extracellular space (Lee et al., 2009, 2013; Gurung et al., 2011; Im et al., 2017; Askarian et al., 2018).

In the study of Askarian et al. (2018) the conclusion is drawn, that EVs produced by *S. aureus* are covered by peptidoglycan (PGN) (or peptidoglycan precursors). This assumption stems from observations in Transmission Electron Microscopy (TEM) experiments. Here, the staining of EVs with an antibody detecting *S. aureus* PGN followed by an immunogold-labeled protein A molecule (Spa-gold) led to the deposition of gold particles on the surface of EVs.

However, this result needs to be interpreted with care. *S. aureus* produces two independent immunoglobulin-binding proteins. Best known is *S. aureus* protein A (Spa). The protein is a member of the cell wall-anchored proteins (CWAs) and the mature protein is anchored to the peptidoglycan by Sortase A (Foster et al., 2014). In addition, *S. aureus* produces the second binding protein for immunoglobulins (Sbi). Sbi does not contain a Sortase A recognition motif (LPxTG) but interacts with lipoteichoic acid, facilitating the localization of Sbi within the bacterial membrane (Smith et al., 2011, 2012). In this context, the Ig-dependent deposition of immunogold on the surface of EVs might have two different explanations. Firstly, the primary antibody might indeed recognize *S. aureus-*derived PGN on the surface of the EVs and the Sbi/Spa-proteins of *S. aureus* do not play a role. Secondly, vesicle-associated Spa/Sbi might bind to the primary antibody in an unspecific way leading to gold deposition in the absence of PGN. Importantly, Sbi was found to be associated with the EVs by Askarian and colleagues.

In general, the usage of an immunogold-labeled Spa molecule is an elegant strategy. A vesicle bound IgG molecule should only be recognized by Spa-gold if it binds its target via the Fab-part, thereby displaying the Fc-part to be bound by Spa-gold. If unspecific binding of the primary antibody occurs via the Fc-part, it should not be accessible to the Spa-gold molecule. However, this is a theoretical consideration lacking controls. Unfortunately, the authors did not use a Spa/Sbi double mutant in their experiments to strengthen their hypothesis. At least an unrelated primary antibody, not recognizing *S. aureus* targets, should have been used to demonstrate the necessity of a PGN-specific antibody.

However, the existence of PGN on the surface of the EVs might also be suggested by the fact that Spa and other CWAs (ClfA, IsdA, IsdB) were also found to be associated with the EVs. This finding was independently described also by Gurung et al. (2011). Since CWAs are generally anchored to the PGN and are normally not associated with membranes, this finding can be interpreted as indirect evidence for the association of PGN with EVs. Yet, this hypothesis is also not solid. All CWAs contain a Sec-secretion signal and remain membrane located until linked to the PGN. It seems possible that vesicles budding from a parental cell carry immature CWAs retained within the membrane. It needs to be mentioned, that both Sbi and Spa are found in substantial amounts in culture supernatants (Smith et al., 2012; O'Halloran et al., 2015). For Spa it is known that the “secreted” form harbors an unprocessed sorting signal, indicating that it was not anchored to the PGN prior to its release. It is tempting to speculate that this form of Spa is actually associated with EVs.

A second concern relates to the primary antibody used in the experiments. The antibody is a monoclonal antibody reported to recognize staphylococcal PGN (Abcam ab20002). However, the epitope of this antibody is to my knowledge not defined. As the antibody was most likely raised by stimulation with PGN-extracts, it is unclear whether it indeed binds to PGN (the MurNAc-GlcNAc backbone) or whether it actually binds to PGN-associated proteins such as CWAs, or even to lipoproteins that are frequently cross contaminating PGN isolations (Müller-Anstett MA et al., 2010). As these factors might also be associated with PGN-free vesicles, it raises additional concern about straightforward interpretation of the TEM results.

The hypothesis that EVs might be coated with PGN is interesting, since it could explain the strong immunostimulatory capacity of EVs. Yet, in my eyes the evidence presented is not sufficient to underpin this idea. Several controls will be needed in the future. Most important will be the use of isogenic *spa* and *sbi* deletion mutants. The phenotype of such mutants in the immunogold-labeling experiments will allow better conclusions about the association of vesicles with peptidoglycan. Alternatively, preparations of vesicles could be treated with PGN degrading enzymes (lysostaphin+mutanolysin) cleaving within the pentaglycine cross bridge and within the MurNAc-GlcNAc backbone, respectively. If PGN is indeed associated with the vesicles, and if the epitope of the antibody is indeed PGN, the treatment should prevent immunogold deposition. Yet, it has to be kept in mind that these PGN degrading enzymes would also release CWAs from the PGN raising again concerns as long as the epitope of the antibody is unclear.

In general, it is hard to imagine how an association of PGN with EVs could be explained on the molecular level. There is accumulating evidence that in *S. aureus* EVs are budding of the parental cells in a fashion dependent on the phenol-soluble modulins (Wang et al., 2018) and that turgor pressure together with hydrolytic enzymes, cleaving the PGN, or together with specific channels facilitate their release (Brown et al., 2015). However, how could it be facilitated that PGN stays attached to the EVs after their release? Are there any proteins tethering the PGN-fragments to the membrane? And how might this association look like on the molecular level? Is it a closed mesh of PGN, or a loose association of small fragments?

I believe that these questions need to be addressed if the structure and function of EVs are to be addressed in future experiments.

## Author contributions

The author confirms being the sole contributor of this work and has approved it for publication.

### Conflict of interest statement

The author declares that the research was conducted in the absence of any commercial or financial relationships that could be construed as a potential conflict of interest.
